# Effect of Polyelectrolyte Mono- and Bilayer Formation on the Colloidal Stability of Layered Double Hydroxide Nanoparticles

**DOI:** 10.3390/nano8120986

**Published:** 2018-11-28

**Authors:** Zoltán Somosi, Marko Pavlovic, István Pálinkó, István Szilágyi

**Affiliations:** 1 MTA-SZTE Lendület Biocolloids Research Group, Department of Physical Chemistry and Materials Science, University of Szeged, H-6720 Szeged, Hungary; somosiz@chem.u-szeged.hu (Z.S.); mmpavlovic90@gmail.com (M.P.); 2 Interdisciplinary Excellence Centre, Department of Physical Chemistry and Materials Science, University of Szeged, H-6720 Szeged, Hungary; 3 Material and Solution Structure Research Group, Department of Organic Chemistry, University of Szeged, H-6720 Szeged, Hungary; palinko@chem.u-szeged.hu

**Keywords:** layered double hydroxide, polyelectrolyte layer, colloidal stability

## Abstract

Sequential adsorption of polyelectrolytes on nanoparticles is a popular method to obtain thin films after deposition. However, the effect of polyelectrolyte multilayer formation on the colloidal stability of the nanoparticles has not been studied in detail. In the present work, layered double hydroxides (LDH) were synthesized and interaction with oppositely and like-charged polyelectrolytes was investigated. Electrophoretic and light scattering measurements revealed that colloidal stability of LDH can be tuned by adsorption of poly(styrene sulfonate) (PSS) on the oppositely charged LDH surface in appropriate doses and thus, unstable or stable dispersions can be designed. Negatively charged LDH of adsorbed PSS monolayer was obtained and a poly(diallyldimethyl ammonium chloride) (PDADMAC) second layer was systematically built on the particles. The obtained polyelectrolyte bilayer provided high colloidal stability for the LDH-PSS-PDADMAC dispersions due to the presence of repulsive interparticle forces of electrostatic and steric origin. The results provide crucial quantitative information on designing highly stable particle-polyelectrolyte systems for the preparation of thin films or immobilization of guest substances between the layers for delivery processes.

## 1. Introduction

Self-assembled multilayers have been in the focus of many research groups due to their widespread applications in electrochemical devices, sensors, membranes, smart coatings and biotechnological procedures [1]. In the preparation processes, the sequential adsorption technique is applied, in which polyelectrolytes [2], nanoparticles [3], metal complexes [4], enzymes [5] and other suitable compounds [6,7,8,9] were used in the individual adsorption steps. Different substrates were applied as base for the multilayer formation including both planar surfaces [10] and colloidal particles [11]. In the latter case, once polyelectrolytes are applied as building blocks, the choice of the appropriate dose is critical, since polyelectrolyte adsorption on oppositely charged particles may lead to charge neutralization and unstable particle dispersions containing large aggregates, which are insufficient for further applications [12,13,14]. At higher polyelectrolyte loadings, however, stable dispersions of primary polyelectrolyte coated particles are formed and these samples can be processed well.

Among various nanomaterials, layered double hydroxides (LDH) [15,16,17] are excellent candidates as building blocks in sequential adsorption processes [18,19,20]. LDH are multifunctional materials, therefore, they are widely used in numerous fields including sensing [21], drug delivery [22], catalysis [23,24], electrochemistry [25,26] and environmental processes [27,28]. Due to the positive structural charge of LDH, they were applied together with negatively charged polyelectrolytes such as poly(styrene sulfonate) (PSS) in multilayered compounds. For instance, unilamellar LDH nanosheets were prepared by delamination of the layered structure and assembled layer-by-layer with PSS on glass substrate to obtain composite films of interesting magneto-optical properties [29]. Embedding ruthenium(III) complexes between the LDH-PSS layers led to the development of electrochemiluminescence sensors [4]. Polymer-modified LDH-PSS multilayered films showed excellent oxygen barrier performance with self-healing ability [30]. LDH films were prepared in a hydrothermal process and modified with PSS to achieve higher drug loading ability [31]. The drug release could be smartly controlled from the obtained hybrid materials. In addition, an LDH-PSS-based nanocontainer was synthesized to deliver corrosion inhibitors, which were either embedded in the polyelectrolyte layer or intercalated within the interlayer space of LDH [32].

The applications mentioned above clearly shed light on the importance of the interaction between the LDH particles and the polyelectrolytes. Accordingly, the surface charge properties and aggregation of LDH particles in the presence of oppositely charged polyelectrolytes such as PSS is a key issue to design stable colloids for the preparation of hybrid materials. Apart from its importance, systematic colloid stability studies involving LDH and negatively charged polyelectrolytes were rarely published in the past. As one of the few examples of such studies, adsorption of poly(acrylic acid) on LDH nanosheets led to charge neutralization and to rapid particle aggregation at intermediate doses [33]. Increasing the polyelectrolyte loading in the same system gave rise to the reversal of the sign of particle charge and to stable dispersions. Similar observations were also reported with LDH in the presence of oppositely charged heparin polyelectrolyte [34] and acrylate-based copolymer [35]. However, no results were published concerning the influence of PSS on the charging and aggregation of LDH particles in the corresponding literature.

In the present study, therefore, we report a systematic investigation on the colloidal stability of LDH in the presence of PSS and poly(diallydimethyl ammonium chloride) (PDADMAC) polyelectrolytes. The LDH-PSS systems were prepared by electrostatic adsorption of the polyelectrolyte on the oppositely charged particles, while the LDH-PSS-PDADMAC samples were obtained by adsorbing PDADMAC on the LDH particles of a saturated PSS layer on the surfaces. The effect of polyelectrolyte dose on the colloidal stability and the resistance against salt-induced aggregation were investigated in electrophoretic and light scattering measurements.

## 2. Results and Discussion

### 2.1. Characterization of LDH

**Figure 1:**
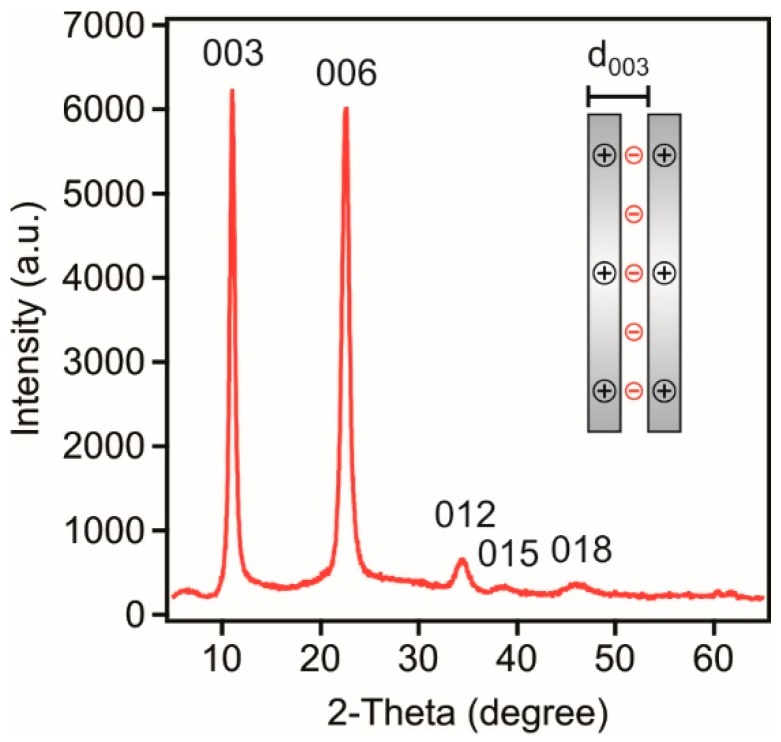
Powder XRD pattern of the LDH particles composed of magnesium(II) and aluminium(III) cations and chloride interlayer anions. The inset illustrates the meaning of the *d*_003_ value calculated from the (003) reflection.

The sheet-like LDH particles composed of magnesium(II) and aluminium(III) layer forming metal ions and chloride intercalated anions were prepared by the flash co-precipitation method followed by hydrothermal treatment to narrow the particle size distribution. The synthetic process is detailed later (see Materials and Methods section). The formation of the lamellar structure was verified by X-ray diffraction (XRD). The diffractogram is shown in Figure 1.

Indeed, the diffraction pattern unambiguously confirmed the formation of the LDH, since the typical Miller indices for LDH could be assigned to the obtained material [20,34,36]. The sharp (003) and (006) reflections indicate the good crystallinity of the lamellar compound. To quantitatively analyze the XRD peaks, the Bragg equation [37] was used to calculate the d-spacing values as

(1) $$n\lambda =2d\sin\theta_{B}$$

where n is an integer (in general it is 1), λ is the wavelength of the incident wave, d is the lattice spacing and $\theta_{B}$ is the Bragg angle. Using the position of the (003) peak, the $d_{003}$ parameter, which represents the distance of one layer together with the interlayer space (see Figure 1 inset), was found to be 0.8 nm. The average size of the ordered domain (υ) was also calculated from the (003) peak with the Scherrer’s equation [38] as

(2) $$\upsilon =K\lambda /\beta \cos\theta_{B}$$

where K is the shape factor (0.89 was used in the calculation) and β is the line broadening at the full widths at half of the intensity. The thickness of the particle was calculated as about 30 nm meaning that the LDH is composed of about 38 layers.

The hydrodynamic radius of the LDH was determined in stable dispersions by dynamic light scattering (DLS) and the measurements yielded (167 ± 6) nm and polydispersity index of (0.23 ± 0.03). Electrophoretic mobility of the particles was measured in the same sample and was found to be (1.0 ± 0.5) × 10^−8^ m^2^/Vs. These values indicate relatively narrow particle size distribution and low surface charge and they are similar to those reported for LDH materials earlier [13,34,35].

### 2.2. Colloidal Stability of LDH in the Presence of PSS

**Figure 2:**
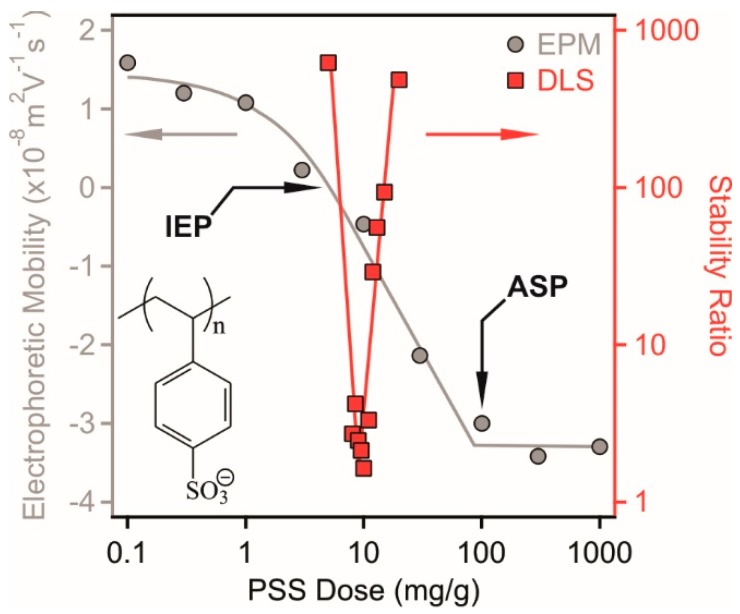
Electrophoretic mobility (circles, left axis) and stability ratio (squares, right axis) values of LDH particles in the presence of PSS polyelectrolyte. The measurements were carried out at 10 mg/L particle concentration and 10 mM NaCl was used as background electrolyte to adjust the ionic strength. The mg/g unit on the x-axis refers to mg PSS per one gram of LDH. The structure of PSS is shown in the inset.

Surface charge properties of the LDH particles in the presence of PSS were assessed in electrophoretic mobility (EPM) measurements. Similarly to other LDH-polyelectrolyte systems [13], the adsorption of PSS is expected to occur on the outer surface of the particles, since intercalation is not feasible due to the narrow interlayer spacing. In the first experiment, the polyelectrolyte concentration was varied in a wide range at constant particle concentration and ionic strength (Figure 2).

Positive EPM values were measured at low PSS doses due to the structural charge of the particles. The data decreased with increasing the polyelectrolyte dose indicating the PSS adsorption on the oppositely charged surface. Such an adsorption process led to charge neutralization at the isoelectric point (IEP), where the overall charge of the particles is zero. Further addition of the polyelectrolytes gave rise to charge reversal and the adsorption continued until the onset of the adsorption saturation plateau (ASP), which corresponds to the dose at surface saturation. The value of ASP was calculated from the intercept of the linear fits on the plateau and in the decreasing mobility regime. This dose was found to be 100 mg/g and the EPM data were constant after this value. In other words, the onset of ASP refers to the maximum amount of PSS, which is able to adsorb on the LDH and further added polyelectrolytes remain dissolved in the solution. Similar charge neutralization and reversal processes were reported in oppositely charged particle-polyelectrolyte systems [12,39,40] including LDH-containing dispersions [13].

The EPM values were measured at different ionic strengths in a wide PSS concentration range (Figure S1) to probe the effect of the ionic environment on the charging properties of the LDH. Similar tendencies were observed at all salt concentrations (1 mM, 10 mM and 100 mM) as discussed above, i.e., charge neutralization at the IEP followed by charge reversal and the adsorption continued until the ASP. However, no clear dependence of the EPM was detected on the salt concentration under these conditions, i.e., the data scattered within the experimental error. It was assumed that such an insensitivity to the ionic strength is due to the counterion condensation into the adsorbed polyelectrolyte chains [41], which prevented efficient screening of the surface charge.

**Figure 3:**
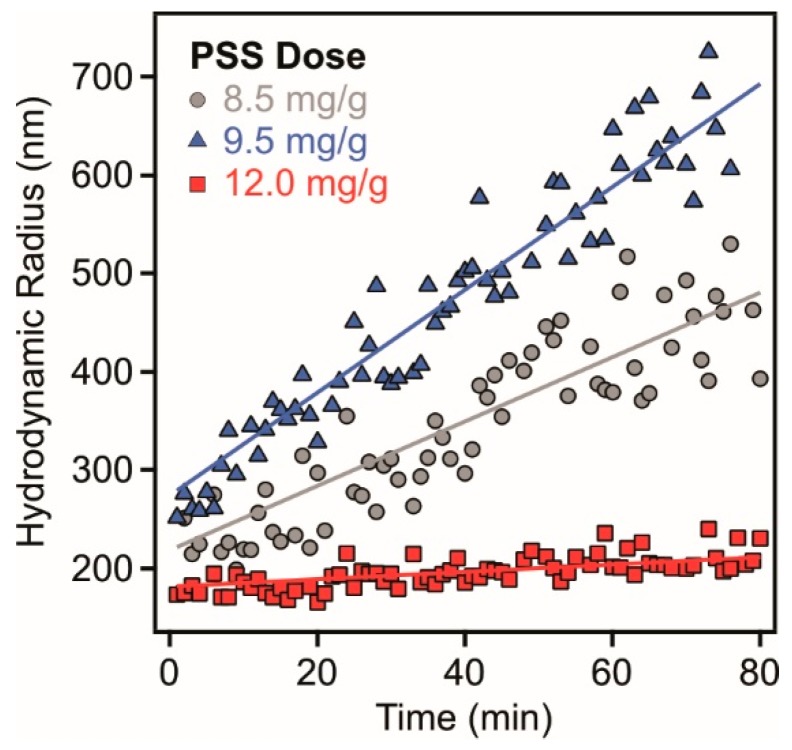
Hydrodynamic radii of LDH particles as a function of the measurement time in the presence of 8.5 mg/g (triangles), 9.5 mg/g (circles) and 12.0 mg/g (squares) PSS. The solid lines are linear fits used to calculate the apparent rates according to Equation (3).

To assess the colloidal stability of the dispersions under the same experimental conditions as applied in the EPM study, stability ratios were determined as follows. Since light scattering techniques have proved as excellent tools to investigate the size of particles and corresponding stability of the dispersions [35,39,40,42,43], time-resolved DLS measurements were carried out, in which the hydrodynamic radius ($R_{h}$) was measured at different time intervals (Figure 3).

In aggregating dispersions, the $R_{h}$ data increased linearly with time (t) indicating that the aggregation processes are in early stages. From the $R_{h}$ versus time plots, the apparent rate ($k_{app}$) of the particle aggregation was calculated and the colloidal stability was expressed in terms of stability ratio (W) as [34,40,42]

(3) $$W=\frac{k_{app}^{fast}}{k_{app}}=\frac{{dR_{h}(t)/dt|}_{t\to 0}^{fast}}{{dR_{h}(t)/dt|}_{t\to 0}}$$

where fast refers to diffusion controlled aggregation, which was achieved in 1 M NaCl solutions. From Equation (3), one can realize that stability ratio value of one indicates rapid particle aggregation and unstable dispersions, while higher values are signals for slower aggregation processes and more stable samples.

The time dependent $R_{h}$ data (Figure 3) recorded at the IEP (9.5 mg/g) as well as below (8.5 mg/g) and above (12.0 mg/g) the IEP indicate that the speed of the aggregation is sensitive to the PSS dose. Indeed, high or not even measurable stability ratios were measured at low and high polyelectrolyte concentrations, where the particles possess positive and negative charge, respectively (Figure 2). Moreover, rapid aggregation of the LDH was experienced at doses close to the IEP indicating unstable dispersions in this regime. However, the location of the fast aggregation regime is slightly shifted comparing to the dose at the IEP due the low charge and the subsequent small velocity of the particles in the electric field in this regime, which leads to uncertainty of the EPM data close to the IEP.

Such a destabilization-restabilization behavior with increasing polyelectrolyte dose was reported for other LDH-polyelectrolyte systems too [13,33,34,35,44] and it can be qualitatively explained by the classical theory of Derjaguin, Landau, Verwey and Overbeek (DLVO) [45,46]. Accordingly, charged particles below and above the IEP dose are stabilized by the repulsion induced by the overlap of the electrical double layers of the particles. At the IEP, however, the LDH possess no charge, i.e., electrical double layer repulsion vanishes and attractive van der Waals forces predominate. Such an attraction causes rapid aggregation of the particles leading to unstable dispersions.

### 2.3. Effect of Polyelectrolyte Bilayer Formation on the Colloidal Stability

**Figure 4:**
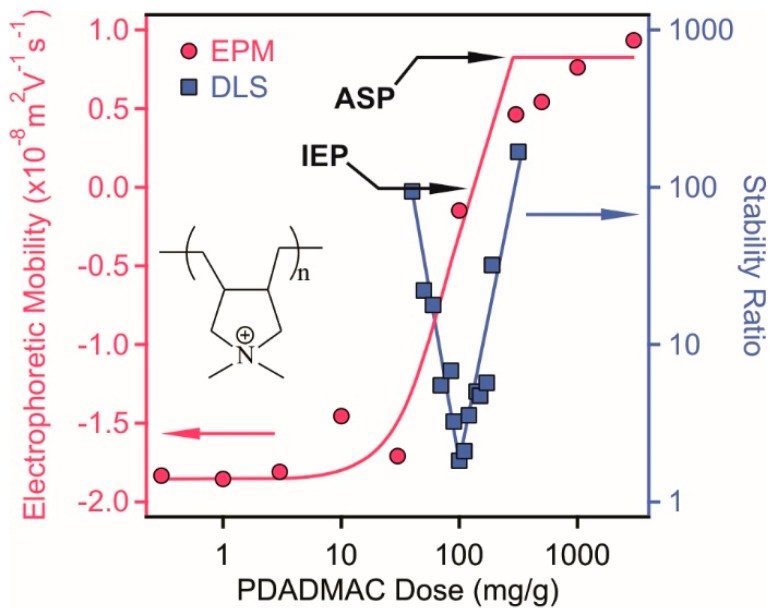
Electrophoretic mobilities (red circles, left axis) and stability ratios (blue squares, right axis) of LDH-PSS (100 mg PSS per one gram of LDH, which corresponds to the dose of the onset of the adsorption saturation plateau (ASP)) as a function of the poly(diallyldimethyl ammonium chloride) (PDADMAC) concentration. The measurements were performed at 10 mg/L particle concentration and 10 mM ionic strength adjusted by NaCl. The structure of PDADMAC is shown in the inset.

As shown above, a saturated PSS monolayer was formed on the LDH at a dose corresponding to the onset of the ASP (denoted as LDH-PSS later) and stable dispersion was observed under this experimental condition. In the next step, a second polyelectrolyte layer was systematically built by adsorbing positively charged PDADMAC polyelectrolyte on the LDH-PSS (100 mg/g of PSS dose, which is equal to the ASP) hybrids at 10 mM ionic strength. Electrophoretic mobilities and stability ratios (Figure 4) were determined first to probe the influence of the PDADMAC concentration on the charging and aggregation properties of the LDH-PSS particles.

Both EPM and stability ratio data followed very similar trend as discussed before with the system containing only LDH and PSS (Figure 3). Namely, charge neutralization and reversal occurred due to the adsorption of PDADMAC on the LDH-PSS. However, the extent of charge reversal is smaller compared to the previous system and the ASP dose was determined as 300 mg/g. The EPM data were measured also at different ionic strengths (1 mM, 10 mM and 100 mM shown in Figure S2) and the EPM values at the ASP decreased with increasing the NaCl concentration due to charge screening. The IEP data were very similar indicating the same adsorption mechanism at different ionic strengths.

The stability ratios could be predicted by the DLVO theory, i.e., fast aggregation near the IEP and stable samples below and above the IEP owing to the presence of repulsive electrical double layer and attractive van der Waals forces. Such a behavior was reported for bare colloidal particles in the presence of PDADMAC [47,48,49,50,51]. To our best knowledge, however, this is the first time, when PDADMAC-induced destabilization-restabilization of polyelectrolyte-functionalized LDH particles is reported. Although comprehensive fundamental studies dealing PSS-PDADMAC multilayers have been published earlier [52,53,54], no information on the stability of colloidal particles functionalized with these polyelectrolytes has been reported.

The hydrodynamic radii of LDH, LDH-PSS and LDH-PSS-PDADMAC were determined in stable dispersions at low ionic strength by DLS (Figure S3) and their values were 167 nm, 216 nm and 352 nm, respectively. The rise in size reflects the formation of the polyelectrolyte layers on the surface. Furthermore, the significant increase for the LDH-PSS and LDH-PSS-PDADMAC compared to bare LDH indicates the presence of an extended polyelectrolyte mono- and bilayer, respectively. The increase is especially large upon adsorption of PDADMAC on LDH-PSS due to the high molecular mass of PDADMAC. In addition, the dispersions are stable before and after the polyelectrolyte addition, but the system passes through the IEP in a transient-like fashion during the adsorption process and becomes unstable for a very short time interval. This small period is long enough for the formation of some aggregates, which leads to certain increase in the hydrodynamic radius [55]. This increment in the size also contributes to the higher hydrodynamic radii for LDH-PSS and LDH-PSS-PDDMAC hybrids.

### 2.4. Resistance against Salt-Induced Aggregation

**Figure 5:**
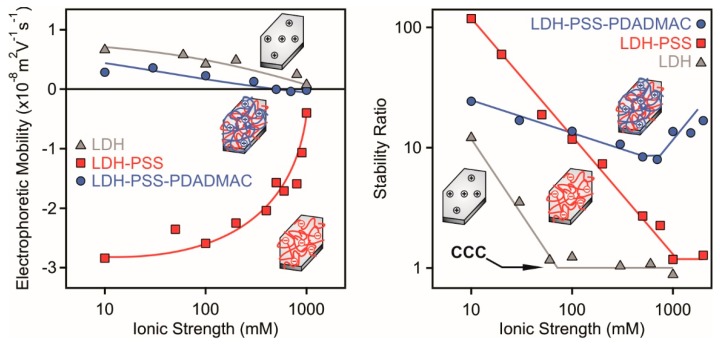
Electrophoretic mobility (left) and stability ratio (right) of bare LDH (triangles), LDH-PSS (squares) and LDH-PSS-PDADMAC (circles) particles versus the ionic strength adjusted by NaCl. A PSS dose of 100 mg/g and PDADMAC of 300 mg/g was applied in the composite particles. These doses correspond to the onsets of the ASP. The insets illustrate an LDH particle and its polyelectrolyte-functionalized derivatives.

One of the limitations of the use of LDH particles in applications taking place in dispersions is their low resistance against aggregation induced by electrolytes, which are present in the majority of the application processes [36]. In general, LDH dispersions are stable at very low ionic strengths, but small amount of salts may cause particle aggregation [13,34,56]. The critical salt level, which separates the stable and unstable regimes, is the so-called critical coagulation concentration (CCC). Polyelectrolyte functionalization has proved to be powerful tool to improve the stability of LDH or other colloidal particles [13,14,35,57,58]. In other words, particles coated with a saturated polyelectrolyte layer possessed higher CCC values than the bare ones. However, systematic studies dealing with polyelectrolyte bilayer-functionalized particles are missing. Therefore, we measured EPM and stability ratio values of LDH-PSS and LDH-PSS-PDADMAC particles (the doses of PSS and PDADMAC in the composites were equal to the onset of the ASP in the individual systems) at different ionic strengths and compared them to the data obtained in the bare LDH system (Figure 5). In this way, the effect of polyelectrolyte layer formation on the resistance against salt-induced aggregation was probed. Note that in these experiments, the polyelectrolyte-modified particles were prepared first in salt-free environment and added to NaCl solutions to obtain the desired ionic strength.

The magnitude of the EPM data decreased with increasing the ionic strength in all cases due to the screening effect of the salt constituent ions on the surface charges (Figure 5, left). Another observation is that the LDH-PSS possessed the highest surface charge indicated by the highly negative EPM values at low salt concentrations. The LDH and LDH-PSS-PDADMAC particles were weakly charged and the mobilities were close to zero at high NaCl concentrations.

The stability ratios of LDH-PSS followed a tendency predicted by the DLVO theory [46] and reported for other polyelectrolyte coated LDH particles earlier [34,35,59]. Accordingly, stable dispersions were observed at low salt levels and stability ratios close to unity indicated rapid aggregation and unstable samples at high ionic strengths. These two regimes are separated by a well-defined CCC (Figure 5, right) at 1000 mM NaCl concentration. Given the low CCC of 50 mM for the bare particles, one can realize that the PSS coating led to an improved resistance against salt-induced aggregation. Such a high CCC cannot be explained by the DLVO theory and an additional stabilizing force should be included in the interpretation. It was assumed that repulsive electrical double layer forces are escorted with steric interactions [33,57,58,60,61], which are also of repulsive nature and originate from the overlapping polyelectrolyte chains on the particle surfaces. Once two particles approach each other, an osmotic pressure is raised leading to repulsive forces. The LDH-PSS dispersions are stable until high ionic strengths, therefore, they can be used in applications taking place in electrolyte solutions.

The behavior of the LDH-PSS-PDADMAC is rather atypical. The stability ratios slightly decrease first with increasing the salt concentration reaching a minimum at around 1000 mM followed by an increase at higher ionic strengths. The stability ratio data are always significantly higher than unity, therefore, the aggregation is slower than in case of the diffusion controlled process. Given the fact that LDH of polyelectrolyte monolayer on the surface can always be destabilized at high ionic strength, the present finding is rather unusual. Moreover, EPM data showed that the particles were moderately charged in the salt concentration regime investigated, therefore, the electrical double layer forces are weak and most probably vanish at high salt levels, where the EPM values are close to zero. The stabilizing forces then must be steric and originate from the adsorbed PSS and PDADMAC chains.

Indeed, it was earlier reported that polyelectrolyte layers on particles swell under certain experimental conditions giving rise to the formation of tails and loops on the surfaces [12,50,55]. This is especially true once the ionic strength is increased in the samples, a condition which leads to thicker polyelectrolyte layers [52,53]. Note that this fact applies for electrostatically adsorbed polyelectrolyte layers, but the opposite trend was reported for polyelectrolyte brushes covalently grafted on surfaces [62,63]. Such a conformation of the polyelectrolytes enhances the development of steric forces due to the overlap between the chains on the approaching particles. Moreover, the hydrodynamic radii in stable dispersions increase significantly with the PSS mono- and PSS-PDADMAC bilayer adsorption (Figure S3) indicating an extended polyelectrolyte layer and bilayer formation on the surface of LDH-PSS and LDH-PSS-PDADMAC particles. Such an extended polyelectrolyte layer enhances the rise of repulsive steric interparticle forces.

## 3. Materials and Methods

### 3.1. Chemicals

The inorganic compounds (MgCl_2_·6H_2_O (VWR, Debrecen, Hungary), AlCl_3_·6H_2_O (Alfa Aesar, Karlsruhe, Germany), NaOH (VWR) and NaCl (VWR)) were of analytical grade. Sodium salt of PSS (molecular mass of 10 kg/mol) and 20 weight % aqueous PDADMAC (average molecular mass of 275 kg/mol) solution were purchased from Sigma-Aldrich (Budapest, Hungary). All chemicals were used without further purification. Ultrapure water produced by a Puranity TU 3 UV/UF+ (VWR) device was used for all sample preparation and measurements. Insoluble impurities from the ultrapure water and from the NaCl solutions were eliminated with a Millex syringe filter of 0.1 µm pore size (Sigma-Aldrich).

### 3.2. LDH Synthesis

The LDH particles containing magnesium(II) and aluminium(III) ions in a 2:1 molar ratio and chloride charge compensating interlayer anions were prepared by the flash co-precipitation method followed by hydrothermal treatment [64]. In the synthetic process, 15 mL of mixed salt solution of magnesium(II) (3.0 mmol) and aluminium(III) (1.5 mmol) chloride was rapidly added into 60 mL of 0.15 M NaOH solution and the mixture was vigorously stirred for 30 min under nitrogen gas atmosphere. The just formed LDH slurry was separated by centrifugation and washed five times with water. The obtained material was then redispersed in 50 mL water. The sample was sonicated for 10 min in an ultrasonic bath and transferred into a stainless steel autoclave with a Teflon lining (Col-Int Tech, Irmo, SC, USA). The autoclave was placed in a preheated oven for a post hydrothermal treatment for 24 h at 120 °C. Thereafter, the autoclave was cooled to room temperature and the LDH dispersion was centrifuged and washed again with water. The LDH was filtered and dried to powder, which was redispersed to obtain a stock dispersion of 10,000 mg/L particle concentration. Some of the powder was kept for XRD characterization.

### 3.3. Light Scattering

Electrophoretic mobility and $R_{h}$ values were measured with a LiteSizer 500 (Anton Paar, Graz, Austria) light scattering device equipped with a 40 mW laser source operating at 658 nm wavelength. All experiments were conducted at 175° scattering angle and at 25 °C. The measurement cells were cleaned with 2 weight % Hellmanex III (Hellma Analytics, Müllheim, Germany) solutions, rinsed with water and dried in a dust-free oven.

Omega-shaped plastic capillary cuvettes (Anton Paar) were used for the electrophoretic experiments. In a typical sample preparation process, calculated amount of ultrapure water, polyelectrolyte and NaCl stock solutions were mixed to adjust the polyelectrolyte concentration and the ionic strength. The total volume of these mixtures was always 1.8 mL. The sample preparation process was finalized by adding 0.2 mL of 100 mg/L LDH stock dispersion. The samples were then left equilibrating for 3 h at room temperature. The capillary cuvettes were flushed with the majority of the dispersions and the last 0.35 mL was used for the actual measurement. The samples were equilibrated for 1 min in the device. The reported electrophoretic mobility values are the average of five individual measurements.

For the DLS experiments, the correlation functions were collected for 20 min and due to the monomodal size distribution of the particles, the Cumulant fit was applied to determine the decay rate constant and the diffusion coefficient [65]. The $R_{h}$ was calculated from the diffusion coefficient using the Stokes-Einstein equation [46]. To determine stability ratios, time-resolved DLS experiments were carried out in plastic cuvettes (Hellma Analytics). The $R_{h}$ values were recorded in different time intervals for 30–60 min depending on the speed of the aggregation. The sample preparation procedure was the same as in the electrophoretic measurements, but the experiments started immediately after adding the particle stock dispersions to the mixed solutions of NaCl and polyelectrolytes.

### 3.4. XRD Measurements

The formation of the layered structure was confirmed by XRD using a PW 1830 diffractometer (Philips, Amsterdam, The Netherlands), which applies Cu-Kα radiation (L = 0.1542 nm) and operates in Bragg-Brentano geometry with Ni filter at a voltage of 40 kV and a current of 30 mA. The diffractograms were recorded in the 4–80° 2-Theta range with a step size of 0.02°. The powder samples were placed on a glass zero background holder for the acquisition of the XRD patterns.

## 4. Conclusions

The present study reports a systematic assessment of the colloidal stability of positively charged LDH particles in the presence of PSS and PDADMAC polyelectrolytes of negative and positive charges, respectively. PSS adsorption led to charge neutralization and subsequent charge reversal at appropriate polyelectrolyte doses and these processes gave rise to destabilization and restabilization of the originally stable dispersions. The aggregation mechanism qualitatively followed the prediction of the classical DLVO theory indicating the presence of interparticle forces of electrostatic origin.

LDH-PSS particles of saturated polyelectrolyte layer on the surface were modified with PDADMAC at different loadings. Increasing the PDADMAC dose resulted in particles of zero overall charge at the IEP and of positive charge at the ASP, where a saturated polyelectrolyte bilayer composed of PSS and PDADMAC was formed on the LDH. The stability of both LDH-PSS and LDH-PSS-PDADMAC particles was investigated in a wide range of ionic strength and it was found that polyelectrolyte functionalization led to an improved colloidal stability of the LDH particles due to repulsion by the electrical double layers and steric interactions between the adsorbed polyelectrolyte chains. The latter effect is stronger, once a PSS-PDADMAC bilayer is formed on the particles.

The results indicate that the use of the LDH can be extended to applications carried out in dispersions containing higher level of electrolytes once the particles are coated with one or two polyelectrolyte layers. The presented sequential adsorption method allows embedding guest molecules between the polyelectrolyte layers in stable dispersions of high specific surface area, since the aggregation and subsequent sedimentation of the LDH is prevented by strong repulsive forces. In addition, the stable LDH-PSS and LDH-PSS-PDADMAC dispersions are excellent candidates as feeding materials in preparation of thin films on appropriate substrates.

## Supplementary Materials

The following are available online at http://www.mdpi.com/2079-4991/8/12/986/s1, Figure S1: Electrophoretic mobility data of LDH in the pre sence of PSS measured at different ionic strengths; Figure S2: Electrophoretic mobility data of LDH-PSS in the presence of PDADMAC measured at different salt concentrations; Figure S3: Hydrodynamic radii of bare and polyelectrolyte-functionalized LDH.
