# Supplementary material for: Effect of Polyelectrolyte Mono- and Bilayer Formation on the Colloidal Stability of Layered Double Hydroxide Nanoparticles
Source: Nanomaterials (Basel). 2018 Nov 28;8(12):986. doi: 10.3390/nano8120986 (PMC6316193; doi:10.3390/nano8120986)
Supplement: Supplementary file 1 [file nanomaterials-08-00986-s001.pdf]

Supplementary Materials

for

Effect of Polyelectrolyte Mono and Bilayer Formation on the Colloidal Stability of Layered Double Hydroxide Nanoparticles

Zoltán Somosi,[1,2] Marko Pavlovic,[1] István Pálinkó[3] and István Szilágyi[1,2,*]

[1] MTA-SZTE Lendület Biocolloids Research Group, Department of Physical Chemistry and Materials Science, University of Szeged, H-6720 Szeged, Hungary

[2] Interdisciplinary Excellence Centre, Department of Physical Chemistry and Materials Science, University of Szeged, H-6720 Szeged, Hungary

[3] Material and Solution Structure Research Group, Department of Organic Chemistry, University of Szeged, H-6720 Szeged, Hungary

* Correspondence: szistvan@chem.u-szeged.hu (I.S.)

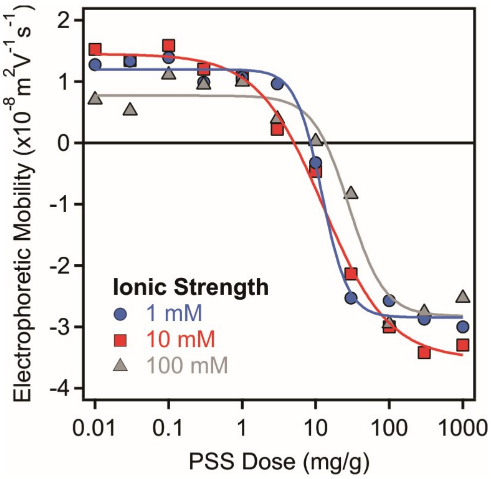

Figure S1. Electrophoretic mobilities of LDH particles as a function of the PSS dose at 1 mM (circles), 10 mM (squares) and 100 mM (triangles) ionic strengths adjusted by NaCl. The measurements were carried out at 10 mg/L particle concentration. The mg/g unit on the x-axis indicates mg PSS per one gram of LDH. The lines serve to guide the eyes.

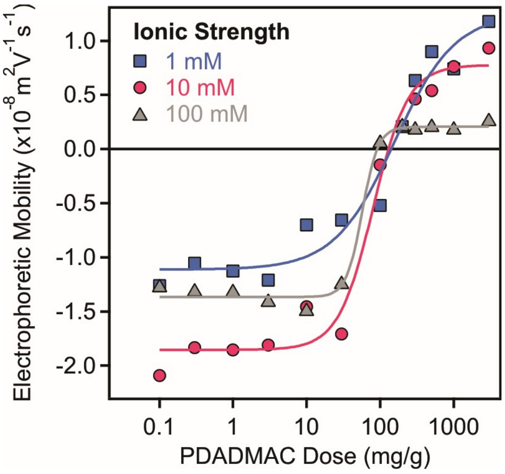

Figure S2. Electrophoretic mobilities of LDH-PSS particles as a function of the PDADMAC dose at 1 mM (squares), 10 mM (circles) and 100 mM (triangles) ionic strengths adjusted by NaCl. The measurements were carried out at 10 mg/L particle concentration. The mg/g unit on the x-axis indicates mg PDADMAC per one gram of LDH-PSS. The lines serve to guide the eyes.

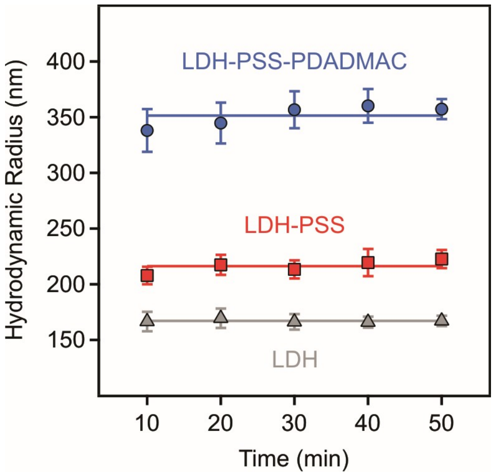

Figure S3. Hydrodynamic radii of bare LDH (triangles), LDH-PSS (squares) and LDH-PSS-PDADMAC (circles) particles versus time at 3 mM ionic strength. Each data point is the average of 10 hydrodynamic radii measured in time-resolved DLS experiment. A PSS dose of 100 mg/g and PDADMAC of 300 mg/g was applied in the composite particles. The solid lines are linear fits, where the slope was set to zero.
